# Stress Heterogeneities in Sheared *Type-I* Collagen Networks Revealed by Boundary Stress Microscopy

**DOI:** 10.1371/journal.pone.0118021

**Published:** 2015-03-03

**Authors:** Richard C. Arevalo, Pramukta Kumar, Jeffrey S. Urbach, Daniel L. Blair

**Affiliations:** Department of Physics and The Institute for Soft Matter Synthesis and Metrology, Georgetown University, Washington, DC, USA; University of Cambridge, UNITED KINGDOM

## Abstract

Disordered fiber networks provide structural support to a wide range of important materials, and the combination of spatial and dynamic complexity may produce large inhomogeneities in mechanical properties, an effect that is largely unexplored experimentally. In this work, we introduce *Boundary Stress Microscopy* to quantify the non-uniform surface stresses in sheared collagen gels. We find local stresses exceeding average stresses by an order of magnitude, with variations over length scales much larger than the network mesh size. The strain stiffening behavior observed over a wide range of network mesh sizes can be parameterized by a single characteristic strain and associated stress, which describes both the strain stiffening regime and network yielding. The characteristic stress is approximately proportional to network density, but the peak boundary stress at both the characteristic strain and at yielding are remarkably insensitive to concentration.

## Introduction

Unlike homogeneous continuous solids, disordered granular assemblies show heterogeneous propagation of externally applied stresses along localized linear chains [1]. Similarly, disordered networks of stiff or semi-flexible filaments display unusual mechanical properties [2, 3], including dramatic stiffening when sheared [4]. Recent findings offer a variety of explanations for strain stiffening in network gels. Numerical simulations of deformed biopolymer networks in two- and three-dimensions suggest several potentially interrelated stiffening mechanisms: bending-to-stretching fiber transitions [5–7]; floppy mode responses [8]; nonaffine-to-affine crossover in network rearrangements [9]; fiber buckling [10], directionality [11, 12], and stiffening [4, 13]; and localized force chains pervading the network [14]. Strain-stiffening responses show considerable sensitivity to local topographical features including fiber diameter [15], contour length [16], and branching geometry [17, 18], suggesting that, despite its generality, a complex interplay of phenomena may play a role in the strain stiffening process. An in depth discussion of recent modeling advances can be found in [19].

In this work we investigate the heterogeneous nonlinear mechanics of *Type-I* collagen gels. *Type-I* collagen is a major component of the extracellular matrix [20], and purified *Type-I* collagen gels are used to simulate three-dimensional microenvironments [21] for cell motiltity and traction force studies [22, 23] and as a model biopolymer gel [12, 24–26]. Using Boundary Stress Microscopy, we are able to show that the stresses in sheared collagen gels are inhomogenous over length scales much larger than the network mesh size and that the evolution of the stress distribution is consistent with a stiffening and yielding response that is determined by a subset of collagen fibers that reorient and stretch in response to applied shear.

## Materials and Methods

### 0.1 Polyacrylamide gel

**Fig 1:**
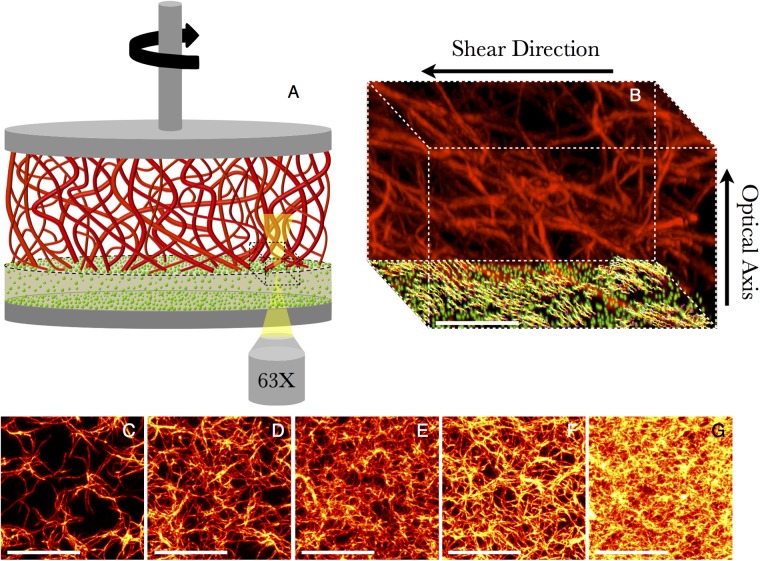
Confocal Rheology system and *Type-I* collagen morphology for all concentraions. (A) An illustration of a collagen network (*red*) adhered to a PAA gel substrate (*tan*) embedded with fluorescent microspheres (*green*) enclosed in a confocal-rheometer (*gray*). Microspheres embedded at the interface (*dotted circle*) are imaged and tracked for calculation of boundary stresses. (Figure not drawn to scale.) A portion of the confocal volume (*dotted box*) is shown in (B), with fluorescently-labeled collagen (concentration *c* = 0.25 mg/mL) and vectors representing the measured microsphere displacements (*γ*_*R*_ = 36%.) Scale bar = 25 *μ*m. (C)-(G) Confocal fluorescence images of collagen networks for concentrations *c* = {0.25, 0.50, 0.75, 1.0, 1.5}mg/mL and mesh sizes *ξ* = {12.1 ± 1.0, 6.9 ± 0.9, 4.8 ± 1.0, 3.8 ± 1.0, 2.2 ± 0.7} *μ*m. Each image is a 10-*μ*m-thick maximum-projection along the optical axis of the collagen network ≈ 20 *μ*m above the PAA gel surface. Scale bar = 25 *μ*m.

Polyacrylamide gels are prepared with a 25-*mm*-diameter and 29 ± 1 *μ*m thickness using a 5%/0.04% acrylamide-to-bisacrylamide concentration ratio (Bio-Rad Laboratories, Hercules, CA) and adhered to glutaraldehyde-activated (Sigma-Aldrich, St. Louis, MO) #1.5 40-mm-diameter glass coverslips (Warner Instruments, Hamden, CT) following published protocols [27] (Fig. 1A, *tan*). Substrates are embedded with sonicated 0.5-*μ*m-diameter carboxylate-modified and bovine-serum-albumin-coated (Sigma-Aldrich, St. Louis, MO) yellow-green fluorescent microspheres (Invitrogen, Eugene, OR) at a 1:10 dilution resulting in an average area per sphere of $8.2\pm 0.3\;\frac{\mu {\text{m}}^{2}}{\text{sphere}}$ at the polyacrylamide surface (Fig. 1A, *green*). The substrate-coverslip system is stored in distilled water (Millipore, Billerica, MA) at room temperature to prevent evaporation prior to collagen gel application. The elastic moduli of gels prepared under identical conditions is $G_{P}^{\prime }=354.0\pm 9.8$ Pa (1% oscillatory strains at 1 Hz, MCR-301 Anton Paar, Graz, Austria). We use a Poisson ratio of *ν*_*P*_ = 0.45 [28] to calculate the Young’s modulus for determination of the stresses at the polyacrylamide surface [29].

### 0.2 Collagen gel

*Type-I* rat tail collagen gels (BD Biosciences, San Mateo, CA; 4 mg/mL) are labeled using 1 *μ*M far-red carboxyfluorescein succinimidyl ester fluorescent dye (Biotium, Hayward, CA) in 10x phosphate-buffered saline (Cellgro, Manassas, VA), 1N NaOH (Acros Organics, Geel, Belgium), and 7.5% w/v sodium bicarbonate (Lonza, Walkersville, MD) at pH 7.50±0.06 with ionic strength $I=0.018\;\frac{\text{mol}}{\text{L}}$. The substrate-coverslip system described above is secured inside a custom-designed confocal-rheometer chamber and a 147.2 *μ*L volume of unpolymerized collagen solution is added to and centered on the wet polyacrylamide surface. A 25-mm-diameter parallel-plate steel measuring tool is lowered to a fixed height 340 *μ*m above the coverslip sandwiching the two-layer system (Fig. 1A, *light gray top*). The collagen gels self-assemble *in situ* at 26^°^ *C* for 72 min between the polyacrylamide surface and steel tool to form networks with a 310.8 ± 1.2 *μ*m height (Fig. 1A, *red*). In a humidity-controlled environment, we apply 0.5% oscillatory strains at 1.0 Hz frequency to the two-layer system to ensure the collagen gel has fully polymerized. This procedure produced strong adhesion between the collagen and the polyacrylamide gel, as verified by both bulk rheology (which showed yield stresses and strains comparable to collagen gels withough PAA) and visual inspection (which showed that yielding did not occur preferentially at the collagen-PAA interface, as shown in Supplemental S1 movie). Characteristic mesh sizes for gels prepared under similar conditions are extracted from fiber spacings along pixel rows and columns in thresholded z-resolved planar images [30] acquired using a TCS SP5 point-scanning confocal microscope (Leica, Mannheim, Germany). Measurements of the average pore diameter determined from pore size distributions [31] produce nearly identical mesh sizes (Supplemental S4 Fig.).

### 0.3 Simultaneous confocal and rheological acquisition

We apply rotational shear *γ*_*R*_ to the two-layer system described above with a strain rate $\dot{\gamma_{R}}=0.56\%\;{\text{min}}^{-1}$ and simultaneously image 144.7×144.7×62.7±1.4 *μ*m volumes (Fig. 1A, *dotted box*) spanning the coverslip through 16.6 ± 0.5 *μ*m above the interface (Fig. 1A, *dotted circle*) using a coupled confocal-rheometer [32]. We measure the bulk shear stress system response *σ*_*R*_ while imaging at 1% intervals of the locally applied strain with a 63X water-oil objective 8.33 mm away from the tool center (two-thirds the tool radius) until the imaged network has yielded.

**Fig 2:**
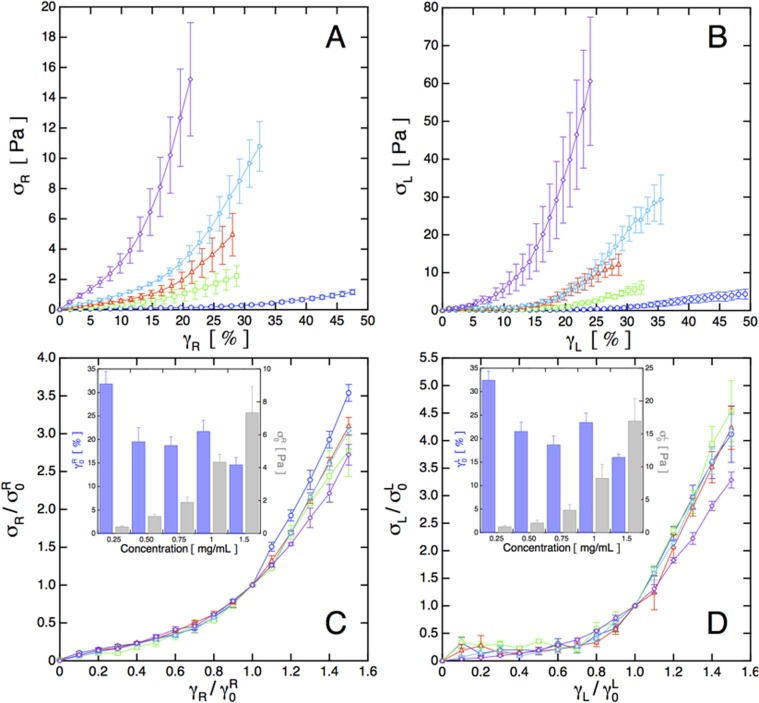
Bulk and local rheology of collagen gels. (A) Bulk stress-strain rheological response of the two-layer system. For all graphs, {∘, □, △, ⊳, ◇} correspond to collagen networks with concentrations *c* = {0.25, 0.50, 0.75, 1.0, 1.5} mg/mL. (B) Average of the local stress measured at the interface with boundary stress microcopy. (C) Rescaling the data in (A) by the stress and strain values at maximum curvature $\sigma_{0}^{R}=\sigma_{R}(\gamma_{0}^{R})$ and $\gamma_{0}^{R}$ (Eq. 3) shows nearly complete data collapse for all concentrations. (D) Rescaling the data in (B) by $\sigma_{0}^{L}=\sigma_{L}(\gamma_{0}^{L})$ and $\gamma_{0}^{L}$ produces a similar data collapse. Scaling parameters for (C) and (D) are shown in (C, inset) and (D, inset), respectively. Error bars represent standard error from {4, 3, 4, 4, 4} trials. All lines connecting points are guides for the eye.

We track the motion of the fluorescent microspheres embedded up to 3 *μ*m below the gel surface and spatially interpolate displacement components projected onto the interface. The resulting deformation maps are spatially low-pass filtered with a 5 *μ*m cutoff and the temporally- and spatially-varying surface stresses at the interface are calculated using an extended traction force technique that inverts the classical Boussinesq solution to the elastostatic equation in Fourier space and modifies the infinite half-space assumption by imposing a zero-displacement boundary condition on the bottom surface of the polyacrylamide layer [29]. This calculation produces all three components of the surface stress at discrete locations on the interface, $\sigma_{x,y,z}({\vec{r}}_{i})$, with the PAA-collagen interface in the x-y plane. We measure the local boundary stress-strain curves *σ*_*L*_(*γ*_*L*_) (Fig. 2B) by calculating the average in-plane surface stress magnitude in the imaged region

$$\begin{array}{c} \sigma_{L}\equiv \left\langle \right\|\vec{\sigma_{∥}}\left({\vec{r}}_{i}\right)\left\|\right\rangle =\langle \sqrt{\sigma_{x}^{2}\left({\vec{r}}_{i}\right)+\sigma_{y}^{2}\left({\vec{r}}_{i}\right)}\rangle \end{array}$$ (1)

where the averages are taken over the positions ${\vec{r}}_{i}$ of each calculated stress map. The local strain is determined according to

$$\begin{array}{c} \gamma_{L}=\frac{G_{P}^{'}\left(h_{C}+h_{P}\right)}{h_{C}G_{P}^{'}+h_{P}G_{C}^{'}}\gamma_{tool} \end{array}$$ (2)

where the $G_{P}^{\prime }$, $G_{C}^{\prime }$, and *h*_*P*_, *h*_*C*_ are the measured linear viscoelastic moduli and thicknesses for the polyacrylamide and collagen gels, respectively, and $\gamma_{tool}=\frac{2}{3}\gamma_{R}$ based on our microscope objective positioning. Note that this formula omits two effects: (i) the nonlinear strain stiffening will produce greater deformation of the PAA layer than predicted by the linear elasticity of the collagen, and (ii) the heterogeneous stress transmission will produce displacements locally that differ from the average. However, in all cases considered here, the PAA layer is considerably stiffer than the collagen, so the variation is small. We have confirmed this by direct measurement of the strain of the PAA layer determined from bead displacements. Deviations from the strain predicted by equation 2 are noticeable only for the highest collagen concentration at the highest strain (when it is stiffest), and even in that regime the deviation is less than 10%.

We determined the minimum detectable local stress of ∼ 2*Pa* from the noise in the stress maps at very low strain, and the minimum resolvable stress peak separation of ∼ 10*μ*m by creating simulated displacement fields from localized forces applied to the surface of an ideal elastic medium randomly sampled at the density of the fiduciary beads in the experiment, and calculating stress maps as with the experimental data.

## Results

The measurement is shown schematically in 1A, and described in detail in the Methods section. Fibrillar collagen networks are polymerized *in situ* between a polyacrylamide gel (bottom) and the rheometer tool (top). The rheometer provides stress and strain control through a rapid feedback between torque and displacement. The motion of fiduciary fluorescent markers in the polyacrylamide (PAA) gels is imaged simulteneously with confocal microscopy, allowing for measurement of the deformation of the PAA-collagen interface in three dimensions (Fig. 1B). Particle displacements are converted into localized three-dimensional surface shear and normal stresses [29]. We have performed measurements over a wide range of collagen concentrations, representing collagen fibril areal densities that range over a factor of 30 (Fig. 1 C-G). Areal densities are determined directly from the measured mesh size, as described in Methods, which is found to scale roughly as *c* ^−1^ (see Supplemental S4 Fig.).

The *bulk* mechanical responses measured by the rheometer *σ*_*R*_(*γ*_*R*_) (Fig. 2A) reveal the signature nonlinear increase in shear stress *σ*_*R*_ with increasing applied strains *γ*_*R*_, consistent with the characteristic stiffening response found in most biopolymer networks [4, 15, 25, 33]. The nonlinear stress increase is associated with a realignment of the underlying network microstructure in the shear direction [12] (S1 Movie). The measured stress-strain curves, including the yield stress (defined as the peak of the stress-strain curves), match those measured without the polyacrylamide layer, indicating that the collagen fibers are effectively bound to the polyacrylamide layers, consistent with direct observation (Movie M1).

The *local* stress-strain curves, *σ*_*L*_(*γ*_*L*_), shown in Fig. 2B represent the average over the 145*μ*m×145*μ*m imaged region, representing less than 0.005% of the total surface area of the rheometer tool. *σ*_*L*_ is determined by averaging the boundary stresses parallel to the PAA-collagen interface calculated from the deformations of the PAA (see methods). The substantial stresses normal to the interface, typical of biopolymer networks [34] and evidenced in Movie M1, are not considered here. The local strain values depend on the radial position of the microscope objective and are adjusted to account for the expected average deformation of the PAA layer. In principle, the *local* deformation may differ from the *average* deformation, and should be determined independently for each measurement. For these results, the deformation of the PAA gel is small, leading to negligible corrections to the value of the local strain (see Methods for details). The local stress-strain curves are qualitatively similar to the bulk response reported by the rheometer with some quantitative differences in magnitude. A direct comparison is complicated by the fact that the rheometer measurement in the parallel plate geometry represents an average over a range of imposed strains that is not easily deconvolved for systems with nonlinear elasticities. Moreover, the rheometer measurement is very sensitive to variations in concentration at the outer radius of the measurement tool. For practical reasons, the collagen gels used in these experiments do not fill the entire measurement volume. Combined with the nonlinear response, these factors can account for the larger stresses determined from the local rheological response.

Despite the quantitative differences, the local and bulk rheological response exhibit similar rheological behavior for all concentrations: a dramatic nonlinear increase of the material stiffness followed by a flattening and eventual decrease (not shown), indicating network yielding. The stiffness, or dynamic modulus, $K(\gamma_{R})\equiv \frac{\delta \sigma_{R}(\gamma_{R})}{\delta \gamma_{R}}$, increases rapidly, reaches a maximum and finally begins to decrease approaching the approximately linear high-strain regime. The applied strain at maximum curvature $\gamma_{0}^{R}$,

$$\begin{array}{c} \gamma_{0}^{R}\equiv \text{Max}\;\left[\frac{dK(\gamma_{R})}{d\gamma_{R}}\right], \end{array}$$ (3)

found by numerically differentiating a smoothed *K*(*γ*_*R*_) response curve, provides a precise, objective measure that we use for a systematic comparison of collagen concentrations.

For each stress-strain curve measured by the rheometer, we rescale *σ*_*R*_ and *γ*_*R*_ by $\sigma_{0}^{R}=\sigma_{R}(\gamma_{0}^{R})$ and $\gamma_{0}^{R}$, respectively (Fig. 2C, inset), to obtain a universal bulk stiffening response curve for all measured concentrations (Fig. 2C). For all concentrations, we observe a universal rheological response, with only slight variations. Similar scaling has been observed in a variety of other systems, including actin [35] and neurofilament [36] networks, synthetic stiff polymers [37], and simulated model networks [38].

Applying the same procedure to the stress-strain curves determined from the local boundary stress measurements, we again find a near complete data collapse; only the highest concentration exhibits a minor deviation from the universal behavior (Fig. 2D). The strain-stiffening is more dramatic in the local stress-strain curves, at least in part because the bulk rheological curves represent a system-wide average over a range of applied strains. While bulk rheological curves measured with a cone-plate geometry can provide uniform strain, they do so with a spatially varying gap, which in collagen also leads to an average over a spatially varying stress [25].

The reference strains shown in the insets of Fig. 2 are roughly independent of concentration, consistent with results in the literature identifying a characteristic strain as the point where the dynamic modulus exceeds the low strain value by a fixed percentage [15, 33]. The increase in *γ*_0_ at the lowest concentration may be attributable to the influence of the finite gap on the measured rheology [25]. The ratio of sample width to the mesh size is ∼ 25 at the lowest concentration, which is approaching the range where finite-size effects were observed to affect measured stress-strain curves, specifically by shifting stresses and yielding to higher strains [25]. By contrast, the values of *σ*_0_ are strongly dependent on concentration, varying by a factor of ∼ 30 over our concentration range. *σ*_0_ is proportional to the measured linear elastic modulus, *G*, as expected from the observed data collapse, which scales roughly as the concentration squared (data not shown), consistent with previous results [33], although the linear rheology of collagen is known to be sensitive to polymerization conditions [39]. Much of the variation in *σ*_0_ can be accounted for by the increase in network density: *σ*_0_ × *ξ* ^2^, which represents the average force per fiber, is roughly constant, varying by less than a factor of 2 (see Supplemental S1 Fig.), suggesting that the microscopic, fiber-level origins of the linear elastic response and strain stiffening are similar at all concentrations. Measurements of the yield stress also scale with *ξ* ^2^ (S1 Fig.), allowing for a concise summary of the local rheological response over the range of concentrations measured: the elasticity is proportional to the areal fiber density, the strain stiffening is a maximum when the average force per fiber is on the order of 100 pN, and the material fails when the force approaches 500 pN. As we show below, however, the applied stress is very unevenly distributed among the fibers.

**Fig 3:**
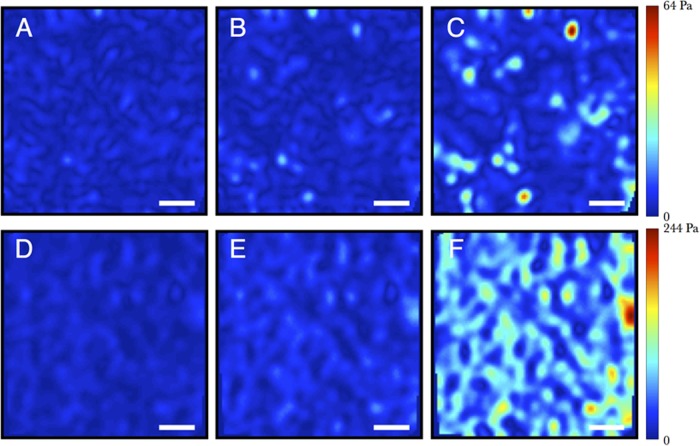
Two dimensional boundary stress dynamics for high and low concentrations. Evolution of stress maps $\left\|\vec{\sigma_{\|}}\right\|$ at the interface for relatively sparse (A)-(C), *c* = 0.50mg/mL and dense (D)-(F), 1.5 mg/mL collagen networks, under applied strains *γ*_*L*_ = {15.1, 18.3, 28.0}% (top row) and {12.9, 16.2, 23.8}% (bottom row), such that $\gamma_{L}/\gamma_{0}^{L}=\{0.8,1.0,1.5\}$ for each row. Scale bar = 25 *μ*m. The dynamic stress maps and associated average boundary stress are shown in the supplemental movies.

**Fig 4:**
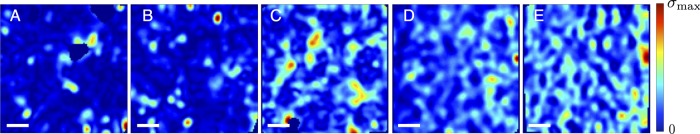
Boundary stresses for all concentrations. (A)-(E) Boundary stresses at $\gamma_{L}/\gamma_{0}^{L}=1.5$ for concentrations c = {0.25, 0.50, 0.75, 1.0, 1.5} mg/mL, respectively, with colormaps normalized by the peak stress in each case. Scale bar = 25 *μ*m.

The evolution of the spatially resolved surfaces shear stresses for two representative concentrations is shown in Fig. 3 and the supplemental movies. At strains significantly below *γ*_0_ the stress in most places does not exceed the noise floor of ∼ 2 Pa. As the applied strain increases, localized high stress regions appear, separated by distances significantly larger than the network mesh size (Fig. 3, A-C and D-F). As the strain increases to near yielding, some additional localized stress peaks appear, while most of the stress maxima present at low strains intensify (Fig. 3 C, F). To more clearly show the changes with network mesh size, the normalized stress maps at *γ*/*γ*_0_ = 1.5 are shown for all concentration in Fig. 4. In all cases the stress distribution is very heterogeneous, with variations on length scales much larger than the network mesh size. At the lowest concentration (Fig. 4A), the peak stresses are separated by large regions of very low stress, with peak stresses more than 10 times the average stress (full stress distributions are shown in the Supplemental S2 Fig.). At the highest concentration (Fig. 4E) the mesh size is less than the spatial resolution, and some of the peaks cannot be cleanly resolved. However, even with this smoothing, the peak stresses are four times larger than the average stress. For the three lowest concentrations, the maximum local stress at yielding is ∼ 100 Pa, independent of concentration (Supplemental S3 Fig.). For the parameters used in the stress calculation, this stress corresponds to an isolated localized force (e.g from a single fiber) of ∼ 4 × 10^−9^ N which is about eight times larger than the average force per fiber estimated from the average stress at yielding. This difference is can be accounted for by the stress heterogeneity, and suggests that it is the tension in the fibers bearing large stresses that are responsible for yielding. At higher concentrations, where the local maxima in the stress maps likely include contributions from multiple fibers, the measured peak stresses at yield increase (S3 Fig.).

Visual inspection of the evolution of the stress maps (S2 and S3 Movies) suggests that the spatial heterogeneity grows with increasing strain, with a rate of increase that slows significantly beyond the point of maximal stiffening *γ*_0_. The minimum detectable local stress of ∼ 2*Pa* limits the dynamic range of the measurement, particularly at low strains and low concentrations. Moreover, the spatial resolution of ∼ 10*μ*m effectively coarse-grains the stress field, so that at the higher concentrations, the contributions of individual fibers blend together. Despite these experimental limitations, the data provide strong support for a model that connects stress heterogeneities and nonlinear strain stiffening. The origin of this connection can be clearly seen in the deformation of the collagen fibers under shear (Movie M1). The strain stiffening regime corresponds to an increasing number of fibers aligned in the direction of the principal stress, and these fibers are responsible for the rapidly increasing, localized stresses. Further increase in strain stretches these aligned fibers, which produces rheology closer to a linear elastic material, with stress increasing approximately linearly with strain, until eventually the contacts can no longer bear the peak forces, which are many times larger than the average force per fiber, and yielding occurs.

While the existence of large stress heterogeneities over length scales much larger than the mesh size indicates that a small fraction of the fibers are bearing the majority of the load, how the load distribution depends on network structure and dynamics remains an open question. Random networks are inherently inhomogeneous, and local variations of mechanical properties can have long-ranged effects [40]. Collagen polymerized *in vitro* shows a complex morphology [18] (Fig. 1) that depends sensitively on polymerization conditions [23]. It is likely that the broad load distribution arises from an interplay between the inherent inhomogeneity of the network and the dynamics of network rearrangement under stress. The stress maps suggests that, at all concentrations, the load is nonuniform over very large scales, and the filaments responsible for bearing the largest load are selected early in the strain stiffening regime and remain engaged until yielding.

The form of stress propagation is central to understanding many important mechanical properties, including the ultimate strength of a material and the nature of appropriate microscopic constitutive equations. Using Boundary Stress Microscopy we directly measure the *local* rheological behavior, rather than averaging over a range of strains or sample widths, and can quantify directly heterogeneous surface stresses. We show that collagen networks exhibit dramatic stress heterogeneity in the strain stiffening regime, and that stiffening and yielding can be understood through the peak forces borne by a subset of the network fibers, which for the networks measure here are an order of magnitude larger than the average forces.

## Supporting Information

S1 Fig Estimated force per fibril. Estimated average force per fibril at the point of maximum curvature in the stress-strain curves (lower curve) and at yielding (upper curve), calculated by multiplying the average of the boundary stress by the square of the mesh size, determined as described in Methods. The forces are surprisingly insensitive to concentration, despite an fibril density that varies by a factor of 30. The somewhat lower value at the highest concentration may be due to the difficulty of acquiring an accurate mesh size measurement for length scales approaching the resolution of optical imaging.
(PDF)

S2 Fig Distribution of surface stresses at the boundary. (A-C) Probability distributions of boundary stress magnitudes at the interface, normalized by the average stress, at strains $\gamma_{L}/\gamma_{0}^{L}=\{0.1,1.0,1.5\}$, respectively. Insets: Histograms of unnormalized stress magnitudes. (A) The distributions at low strains represent the stress noise floor and are therefore independent of concentration. (B-C) At intermediate and large strains the distributions broaden significantly, particularly for the lower concentrations. The finite spatial resolution of Boundary Stress Microscopy, which is comparable to or greater than the mesh size for all concentrations measured, complicates the interpretation of the stress distribution histograms. For all graphs, {∘, □, △, ⊳, ◇} correspond to collagen concentrations c = {0.25, 0.50, 0.75, 1.0, 1.5} mg/mL, respectively. Error bars represent standard error from {4, 3, 4, 4, 4} trials. All lines connecting points are guides for the eye.
(PDF)

S3 Fig Local stress magnitude at maximum curvature. Magnitude of the highest measured value of the local stress at the point of maximum curvature in the stress-strain curves (lower curve) and at yielding (upper curve). At low concentrations, where the distance between stress peaks is greater than the spatial resolution of the boundary stress microscopy, the stress values are relatively insensitive to concentration, unlike the average stresses.
(PDF)

S4 Fig Calculated collagen mesh sizes. Mesh sizes extracted from distributions of fiber spacings along pixel rows and columns in image slices (squares) and from average pore diameter determined from pore size distributions using full three dimensional images (circles). The measurements are completely independent yet yield nearly identical values and similar dependence on concentration. Error bars represent the standard deviation of 16 measurements at each concentration (2 different samples imaged at 8 different places). In all cases, images are binarized with a threshold of 25% of the average intensity.
(PDF)

S1 Movie Rendering of collagen at the gel interface. Rendering of three dimensional movie of a collagen gel at concentration *c* = 0.25 mg/mL between the upper rheometer tool, and the PAA gel. Only the upper surface of the PAA gel is shown. Collagen (red), 0.5*μ*m diameter microspheres in collagen (blue) and 0.5 *μ*m microspheres in PAA (green, see Methods). The collagen is being sheared from right to left to a maximum strain of *γ* = 50% at a strain rate of $\dot{\gamma }=2.8\times 10^{-2}\;\%{\text{s}}^{\text{-1}}$. The collagen remains well attached to the PAA substrate over all strains and clearly deforms the PAA interface.
(MOV)

S2 Movie Dynamic stress map 1. Dynamic stress maps $\left\|\vec{\sigma_{\|}}\right\|$ for *c* = 0.5 mg/mL as described in the main text and Fig. 3(A-C).
(MOV)

S3 Movie Dynamics stress map 2. Dynamic stress maps $\left\|\vec{\sigma_{\|}}\right\|$ for *c* = 1.5 mg/mL as described in the main text and Fig. 3(D-F).
(MOV)
