# Supplementary figures and images for: Stress Heterogeneities in Sheared Type-I Collagen Networks Revealed by Boundary Stress Microscopy
Source: PLoS One. 2015 Mar 3;10(3):e0118021. doi: 10.1371/journal.pone.0118021 (PMC4348423; doi:10.1371/journal.pone.0118021)

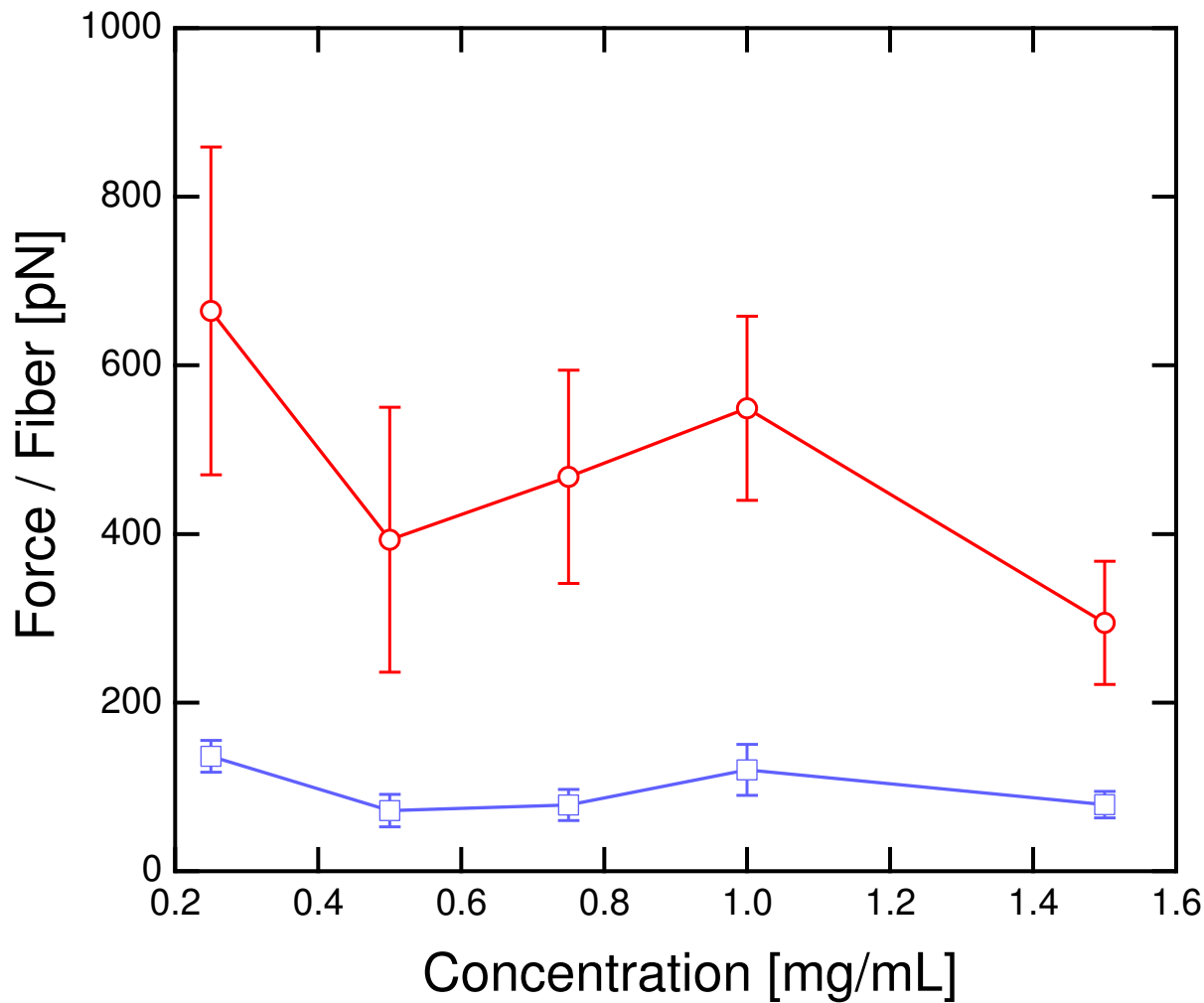

Supplement: S1 Fig — Estimated average force per fibril at the point of maximum curvature in the stress-strain curves (lower curve) and at yielding (upper curve), calculated by multiplying the average of the boundary stress by the square of the mesh size, determined as described in Methods. The forces are surprisingly insensitive to concentration, despite an fibril density that varies by a factor of 30. The somewhat lower value at the highest concentration may be due to the difficulty of acquiring an accurate mesh size measurement for length scales approaching the resolution of optical imaging. (PDF) [file pone.0118021.s001.pdf]

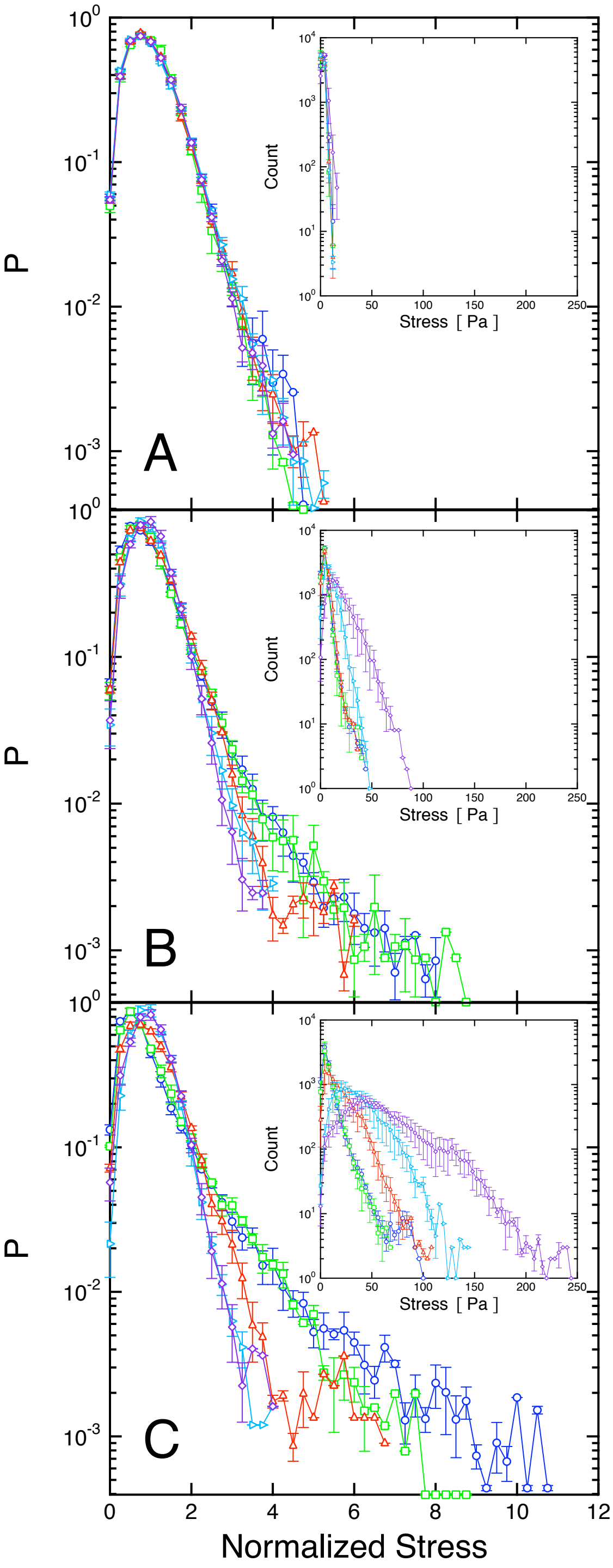

Supplement: S2 Fig — (A-C) Probability distributions of boundary stress magnitudes at the interface, normalized by the average stress, at strains γL/γ0L={0.1,1.0,1.5}, respectively. Insets: Histograms of unnormalized stress magnitudes. (A) The distributions at low strains represent the stress noise floor and are therefore independent of concentration. (B-C) At intermediate and large strains the distributions broaden significantly, particularly for the lower concentrations. The finite spatial resolution of Boundary Stress Microscopy, which is comparable to or greater than the mesh size for all concentrations measured, complicates the interpretation of the stress distribution histograms. For all graphs, {∘, □, △, ⊳, ◇} correspond to collagen concentrations c = {0.25, 0.50, 0.75, 1.0, 1.5} mg/mL, respectively. Error bars represent standard error from {4, 3, 4, 4, 4} trials. All lines connecting points are guides for the eye. (PDF) [file pone.0118021.s002.pdf]

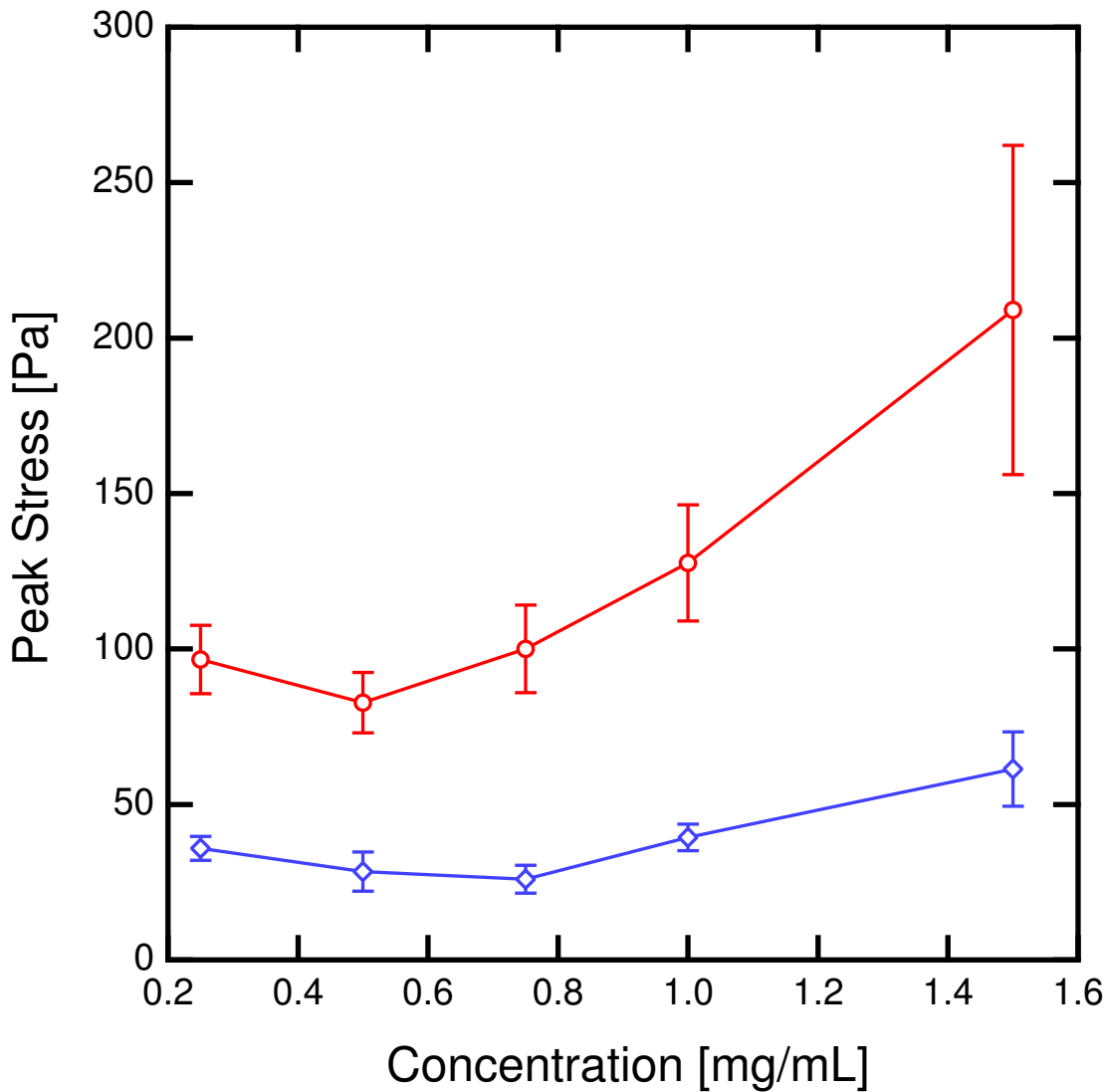

Supplement: S3 Fig — Magnitude of the highest measured value of the local stress at the point of maximum curvature in the stress-strain curves (lower curve) and at yielding (upper curve). At low concentrations, where the distance between stress peaks is greater than the spatial resolution of the boundary stress microscopy, the stress values are relatively insensitive to concentration, unlike the average stresses. (PDF) [file pone.0118021.s003.pdf]

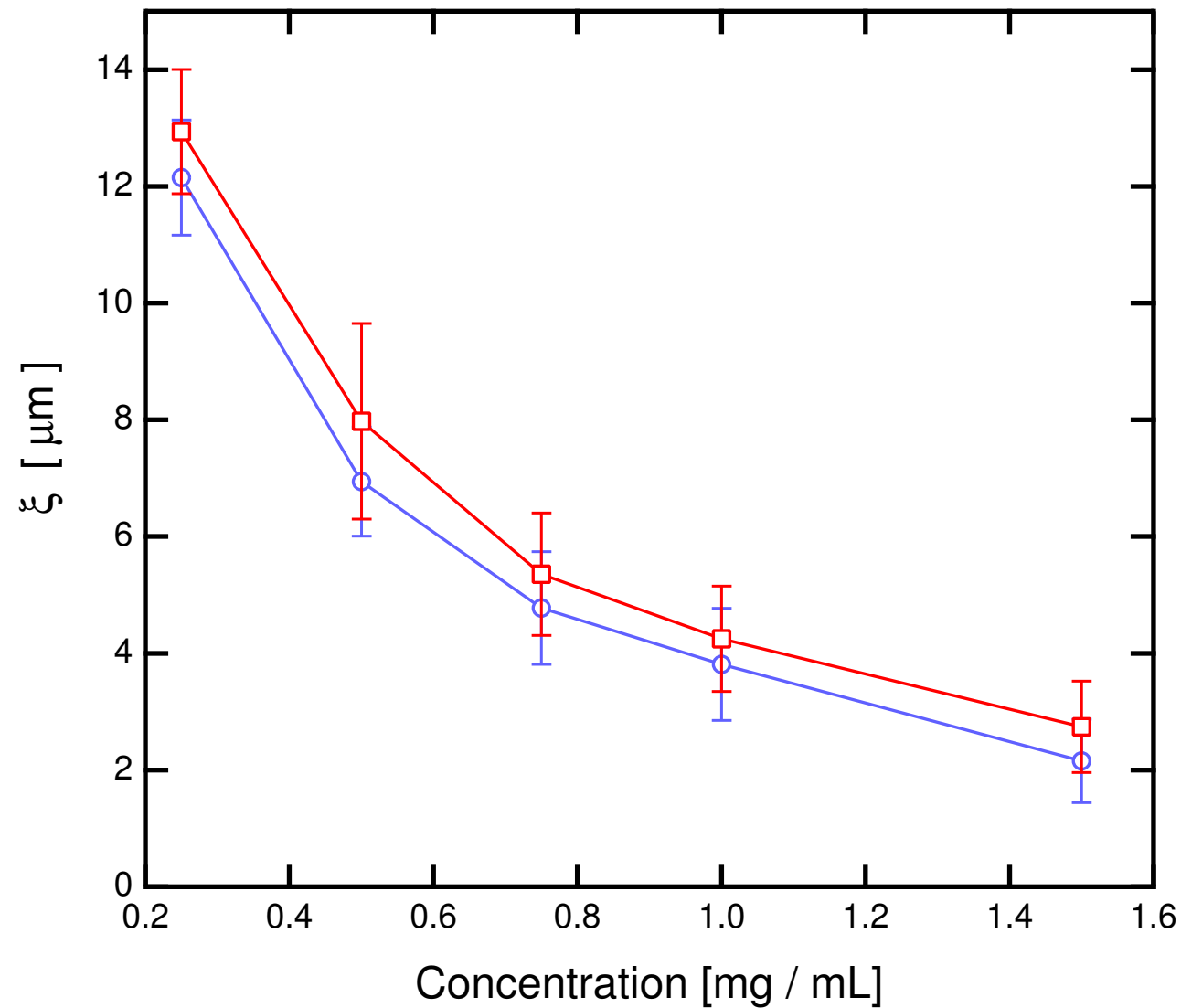

Supplement: S4 Fig — Mesh sizes extracted from distributions of fiber spacings along pixel rows and columns in image slices (squares) and from average pore diameter determined from pore size distributions using full three dimensional images (circles). The measurements are completely independent yet yield nearly identical values and similar dependence on concentration. Error bars represent the standard deviation of 16 measurements at each concentration (2 different samples imaged at 8 different places). In all cases, images are binarized with a threshold of 25% of the average intensity. (PDF) [file pone.0118021.s004.pdf]
